# Evaluation of serum sialic acid level in buffaloes naturally infected with *Theileria annulata*

**DOI:** 10.1007/s11250-016-1096-4

**Published:** 2016-06-16

**Authors:** Bijan Esmaeilnejad, Seyyed Meysam Abtahi Froushani

**Affiliations:** 1 Department of Pathobiology, Faculty of Veterinary Medicine, Urmia University, Urmia, Iran; 2 Department of Microbiology, Faculty of Veterinary Medicine, Urmia University, Urmia, Iran

**Keywords:** Sialic acid, Buffaloe, *Theileria annulata*, Parasitemia

## Abstract

Tropical theileriosis, caused by *Theileria annulata*, is the most economically important disease of domestic buffaloes and causing major losses in livestock production in Iran. Sialic acids are often involved in interaction between the cells and the infectious agents by regulating the molecular relations as well as mediating a variety of cell-cell adhesion processes in the immune response. This study was conducted to assess the effect of *T. annulata* infection on sialic acid concentration in blood sera in naturally infected buffaloes. *T. annulata*-infected (*n* = 22) and uninfected control (*n* = 20) adult buffaloes were selected. *Theileria* infection was revealed by Giemsa-stained peripheral blood and was confirmed by nested-PCR using *T. annulata*-specific primers. Based on the detected parasitemia, the infected animals were subgrouped into low <1 %, moderate 1–3 %, high 3–5 %, and very high >5 %. Hematological parameters and the concentrations of total sialic acid (TSA), lipid-bound sialic acid (LBSA), and protein-bound sialic acid (PBSA) were measured and correlated to parasitemia. The results showed significant differences (*P* < 0.05) in red blood cells (RBCs), packed cell volume (PCV), hemoglobin (Hb), and sialic acid concentrations between infected and control groups. As the parasitemia increased accordingly, a significant decrease in RBCs, PCV, Hb and increase in the mean corpuscular volume (MCV), mean corpuscular hemoglobin concentration (MCHC), and serum sialic acids was observed. We concluded that *T. annulata* infection could elevate the serum sialic acid concentrations. The increased levels of serum sialic acid concentrations during parasitemia presumably stimulate the host immune response and influence the parasite-host cell adhesion.

## Introduction

Hemoparasites of the genus *Theileria* are protozoans which predominantly infect ruminants in tropical and subtropical regions. *Theileria annulata* is the most common species that causes bovine tropical theileriosis in Iran (Nazifi et al. 2009). Tropical theileriosis is a progressive lymphoproliferative disease of cattle and domestic buffaloes characterized by hemolytic anemia (Glass et al. 2003; Asri Rezaei and Dalir-Naghadeh 2006). Economically, the disease imposes heavy losses due to high mortality rates and decreased productivity in affected animals (Ahmed et al. 2002).

Sialic acids (SA), a family of over 40 neuraminic acid derivatives, are among the most important molecules of life, since they occupy the terminal position on macromolecules and cell membranes and are involved in many biological and pathological phenomena (Col and Uslu 2007). The majority of SA is found in either protein bound (PBSA) or lipid bound (LBSA) forms, while a little amount is in the free form. In addition, SA is localized at the end chain of many acute phase proteins (Coskun and Sen 2005; Aytekin et al. 2015).

SA usually occupy exposed terminal positions on the oligosaccharide chains of glycoconjugates and frequently serve as ligands for receptors such as selectins and siglecs, which mediate a variety of cell-cell adhesion processes in inflammation and in the immune response (Malykh et al. 2001). It has been demonstrated that sialic acid concentrations are elevated in patients suffering from various diseases (Citil et al. 2004; Thrall 2004). Although, similar studies are available about sialic acids alteration in bovine and ovine piroplasmosis (Razavi et al. 2010; Esmaeilnejad et al. 2014), but there are no published reports on sialic acids change associated with tropical theileriosis in buffaloes. Therefore, the present study was aimed to assess the alterations of serum sialic acids in buffaloes naturally infected with *T. annulata*. In addition, relationship between serum sialic acid changes and parasitemia has been evaluated.

## Material and methods

### Animals

This study was carried out in the northwest region of Iran (West Azerbaijan Province), where *T. annulata* is prevalent during the summer season (June–September). Diseased group comprised 22 adults water buffaloes, 2–3 years old, naturally infected with *T. annulata*, were divided into four subgroups according to their parasitemia (low <1 %, moderate 1–3 %, high 3–5 %, very high >5 %). These buffaloes had a history of tick infestation, anorexia, prolonged listlessness, increased rectal temperature, dyspnea, tachycardia, pale mucous membrane, and stage of anemia. As a control group, 20 *T. annulata* negative and apparently healthy buffaloes reared under the same farm management and environmental conditions were also sampled. The animals (diseased and control) had not been treated for disease prior to sampling. This study was approved by the Ethical Committee of the Faculty of Veterinary Medicine, Urmia University, Urmia, Iran.

### Blood sampling and parasitological examination

From diseased and control buffaloes, jugular blood samples were taken into vacutainers (KendallCompany, Covidien, USA) containing EDTA-K2 as anticoagulant for determination of hematological and molecular analyses and without anticoagulant for measuring of serum sialic acids concentration. The sera were separated by centrifugation at 750 g for 15 min and stored at −20 °C until use. Ear vein thin blood smears were made, fixed by absolute methanol (5 min), stained with 10 % Giemsa solution (30 min), and examined under oil immersion (×1000) to observe intraerythrocytic forms of *T. annulata*. After examining more than 100 microscopic fields, the parasite was quantified and expressed in percentage of infected erythrocytes (Shiono et al. 2003). The examined smear was recorded as negative if no parasites detected in 200 fields. The positive and negative results were further confirmed by species-specific nested-PCR assay.

### Molecular analysis

DNA was extracted using a DNA extraction kit (Cinagen, Iran) according to the manufacturer’s instructions. DNA was eluted and stored at −20 °C until used. To confirm *T. annulata* infectivity and to rule out the infection in the test and control animals, respectively, nested-PCR was performed as previously described (Martin-Sanchez et al. 1999).

### Hematological examination and measurement of sialic acid

Hemoglobin (Hb) concentration, erythrocyte count, and packed cell volume (PCV) were determined by automated hematology analyzer (Autolyser, Al 820, Swiss). Mean corpuscular volume (MCV) and mean corpuscular hemoglobin concentrations (MCHC) were calculated (Schalm et al. 1975). The concentrations of serum total sialic acid (TSA) (Warren 1959) and LBSA (Karapehlivan et al. 2007) were determined. The amounts of total and LBSA were determined by using a standard sample of *N*-acetyl neuraminic acid (Malykh et al. 2001). PBSA was measured by subtracting TSA from LBSA. Concentration of serum sialic acid in serum samples was expressed in micromoles per milliliter.

### Statistical analysis

Statistical analysis of the ranks was performed with Kruskal–Wallis test followed by pair-wise comparisons using the Mann–Whitney *U* test with Bonferroni adjustment. The relationship among serum sialic acid, parasitemia, and anemia was assessed by linear regression analysis using Statistical Package for the Social Sciences (SPSS, Version 17, Chicago). Values of *P* < 0.05 were considered significant.

## Results

### Parasitemia assessment and molecular confirmation of *T. annulata* infection

The level of parasitemia ranged from less than 1 % to more than 15 %. Of the 22 diseased animals, 5 (22.8 %) had a low level of parasitemia, 7 (31.9 %) moderate, 6 (27.2 %) high, and 4 (18.1 %) very high parasitemia. All of the diseased animals were positive by nested-PCR (an expected 453 bp fragment). On the contrary, there was no amplification of *T. annulata* DNA from the control animals.

### Hematological findings

The mean values of hematological parameters in the control and diseased animals with different parasitemia are presented in Table 1. As the parasitemia rate increased in diseased animals, a significant decrease (*P* < 0.05) was observed in red blood cells (RBCs), PCV, Hb. In contrast, with increase in parasitemia, a significant increase (*P* < 0.05) in MCV and MCHC was evident.

**Table 1:** Mean ± SEM of hematological parameters in control buffaloes and those infected with *T. annulata*

	No	Parasitemia rates	RBCs (10^6^μL^−1^)	PCV (%)	Hb (g dL^−1^)	MCV (fL)	MCHC (g dL^−1^)
Control	20	0	6.4 ± 0.04^a^	29.13 ± 0.1^a^	9.7 ± 0.11^a^	25.2 ± 0.6^e^	22.2 ± 0.1^e^
Diseased	5	Low	6.11 ± 0.02^b^	26.01 ± 0.3^b^	8.1 ± 0.2^b^	29.2 ± 0.1^d^	25.1 ± 0.1^d^
	7	Moderate	5.74 ± 0.01^c^	20.11 ± 0.1^c^	5.7 ± 0.03^c^	35.3 ± 0.2^e^	28.3 ± 0.3^c^
	6	High	4.96 ± 0.06^d^	14.4 ± 0.4^d^	3.6 ± 0.07^d^	42.5 ± 0.1^b^	31.0 ± 0.1^b^
	4	Very high	3.83 ± 0.03^e^	9.6 ± 0.2^e^	2.2 ± 0.2^e^	45.5 ± 0.5^a^	33.2 ± 0.2^a^

Different superscript in each column denotes significant differences (*P* < 0.05)

*SEM* standard error of mean, *RBCs* red blood cells, *PCV* packed cell volume, *Hb* hemoglobin, *MCV* mean corpuscular volume, *MCHC* mean corpuscular hemoglobin concentration

### Measurement of sialic acid

**Fig. 1:**
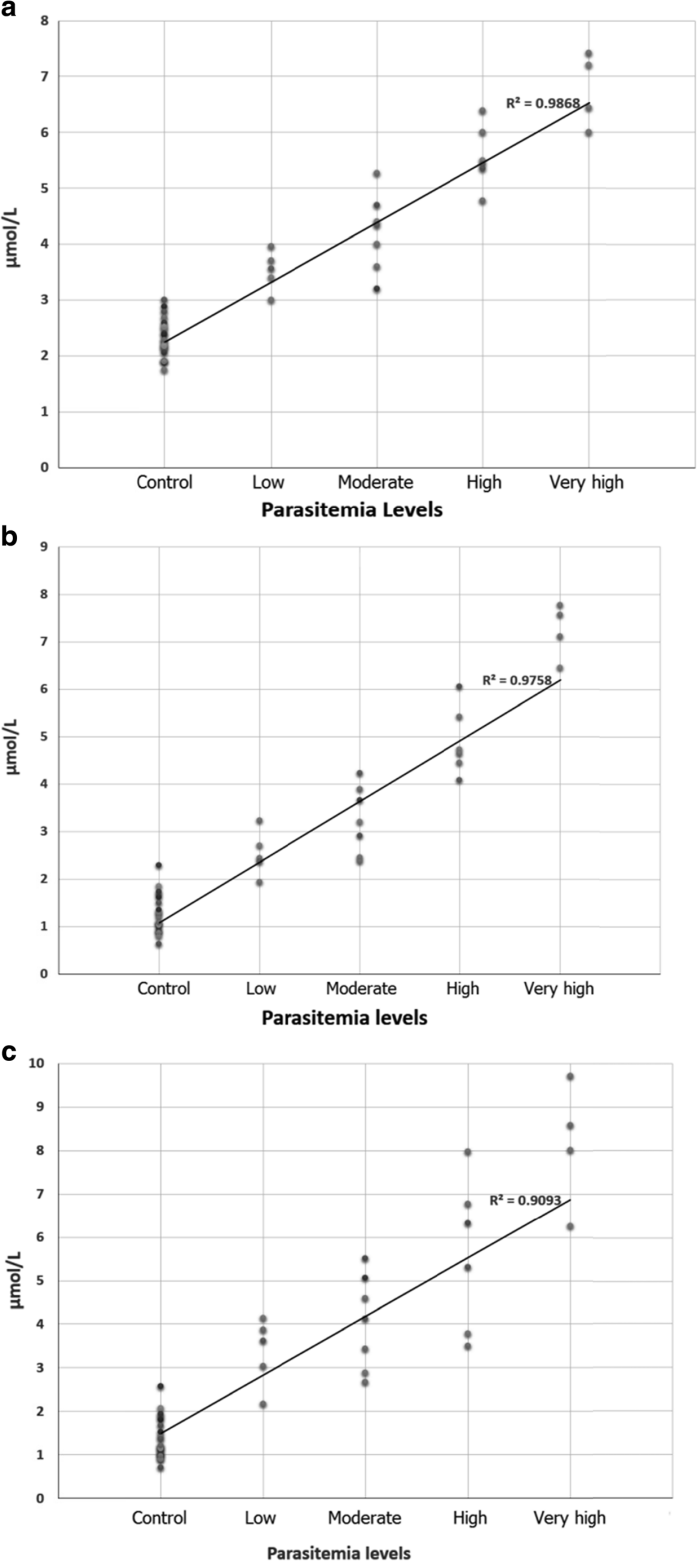
Correlation among different levels of parasitemia and total sialic acid (**a**), lipid-bound sialic acid (**b**), and protein-bound sialic acid (**c**)

The alterations of sialic acid (TSA, LBSA, and PBSA) concentrations in the control and diseased animals with different parasitemia are shown in Fig. 1. A significant increase (*P* < 0.05) in sialic acid concentration was evident in diseased animals. In addition, sialic acid concentrations showed a positive correlation with different levels of parasitemia.

## Discussion

When animals survive to *T. annulata* infection, recovery is extended and is often incomplete, resulting in loss of productivity and a carrier state (Glass et al. 2003). This study was conducted to assess the alterations of serum sialic acids and to find a possible relationship between serum sialic acid changes and parasitemia in buffaloes suffering from tropical theileriosis. Our findings showed that changes in serum sialic acids content may be involved in the pathogenesis of theileriosis caused by *T. annulata*.

In the present study, the RBCs, PCV, and Hb content were significantly lower in diseased animals compared to healthy ones (*P* < 0.05). Also, on progression of parasitemia, a significant decrease in RBC, PCV, and Hb was observed (*P* < 0.05). Therefore, the anemia increases concurrent with the parasitemia rates progression. These findings are in accordance with those of (Hasanpour et al. 2008; Razavi et al. 2010). According to Singh et al. (2001) the protozoan is responsible for the occurrence of anemia, since the lysis of RBC due to multiplication of piroplasms in RBC is one of the main causes of red cell injury resulting in cell destruction.

Concerning the erythrocyte indices, as parasitemia increased, a significant elevation was observed in MCV and MCHC that indicated macrocytic-hyperchromic anemia. Macrocytosis and polychromatophilic erythrocytes in blood smears pointed out a regenerative anemia in diseased animals. Regenerative anemia occurred together with an increase in the count of reticulocytes, which suggests that the elevation of MCV and the increase in MCHC may be due to extravascular hemolysis of infected erythrocytes. Stockham et al. (2000) and Razavi et al. (2010) found macrocytic-hyperchromic anemia in cattle infected with *Theileria buffeli* and *T. annulata*, respectively. Although various evidence has been presented to explain the mechanism of anemia in tropical thieleriosis (Grewal et al. 2005; Chen et al. 2009; Saleh et al. 2011), the exact underlying mechanism(s) of anemia and the complex processes involved is currently unknown.

In the present study, the serum sialic acid concentrations (TSA, LBSA, and PBSA) in buffaloes with tropical theileriosis having different parasitemia were higher than those in control animals (*P* < 0.05). It is currently unclear how infection with *T. annulata* leads to increase the level of serum sialic acid content in buffaloes, but previous studies on ruminant theileriosis indicated that the increased level of serum sialic acids may alter receptor-ligand interactions, which are known to play an important role in inflammation immune response (Kelm and Schauer 1997). Similar to these results, Karagenc et al. (2005) reported the increased levels of serum sialic acid in *T. annulata*-infected animals, whereas Yurtseven and Uysal (2009) reported a significant decrease in serum sialic acid levels of naturally infected cattle with high parasitemia (50–70 %) of *T. annulata*. It seems that these two contradictory results may be due to acute and chronic theileriosis with different percentage of parasitemia seen in young and older cattle, which were analyzed separately in two different studies. In fact, a decrease in concentrations of sialic acid in high levels of parasitemia may be due to utilization of sialic acids during attachment, tight junction, and invasion process of parasites to the host RBC.

According to the previous studies (Kelm and Schauer 1997; Malykh et al. 2001; Karagenc et al. 2005), it can be concluded that sialic acid could modulate biological cell-cell interactions in two nonmutually exclusive ways. First, sialic acid could mask the underlying sugar chains (i.e., lactosaminic sequences), hindering then from interacting with galactose-specific lectins (galectins) (Yurtseven and Uysal 2009). Second, sialic acid would also directly interact with specific sialic acid-binding lectins (siglecs) (Razi and Varki 1998). Therefore, increased contents of sialic acid would interfere with the attachment of sporozoites on host cells, or promote the invasion of erythrocytes by merozoites. Furthermore, the release of sialic acid from the glycolipids or glycoproteins of the lysed cell-membrane surfaces may result in the elevation of serum LBSA, PBSA, and TSA levels (Dall’Olio 2000; Citil et al. 2004).

Infection with *T. annulata* stimulates various immune responses and particularly natural killer (NK) cells which are known to play an important role in the innate immunity to *T. annulata* infection (Guzel et al. 2008). Indeed, NK cells lyse schizont-infected cells and produce interferon gamma (IFN-γ) which activates uninfected macrophages to produce tumor necrosis factor alpha (TNF-α), interleukin-1(IL-1), IL-6, and nitric oxide (NO) (Ahmed et al. 2008). But cell surface hypersialylation hides some antigens, decreasing infected cell susceptibility to NK cells consequently promoting the evasion of the immune response and persistence of parasite in the host. Increased contents of sialic acid would alter receptor-ligand interactions between the sialic acid and its receptors such as selectins and siglecs, which are known to play important roles in the inflammation and in the immune response (Suzuki 1995).

To summarize, this study revealed that natural infection of buffaloes with *T. annulata* leads to significant increases of total serum sialic acid concentrations and that this factor could influence the parasite-host cell adhesion, but determination and explanation of the exact role of sialic acid in invasion process of parasites on host cells and origin of the increase of the serum sialic acids are required more molecular and biochemical investigations.
